# Supplementary material for: Evaluating the Feasibility and Acceptability of a Prototype Hospital Digital Antibiotic Review Tracking Toolkit: A Qualitative Study Using the RE-AIM Framework
Source: Antibiotics (Basel). 2025 Jul 1;14(7):660. doi: 10.3390/antibiotics14070660 (PMC12291640; doi:10.3390/antibiotics14070660)

‘Evaluating the Feasibility and Acceptability of a Prototype Hospital Digital Antibiotic Review Tracking Toolkit: A Qualitative Study Using the RE-AIM Framework’

Table of contents

Interview Topic Guide 2

Consolidated criteria for reporting qualitative studies (COREQ).................... 4

DARTT Components Summary.................... 9

Coding Index....................12

Indexing Example....................13

Charting....................18

Mapping and Interpretation....................19

Interview Topic Guide

Introduction:

- Thank you for seeing me today and for agreeing to take part in this study.
- I would like first to outline the study so that you are able to decide whether you wish to proceed further (recap Information Sheet).
- Sign consent forms (1 for participant and information sheet, 1 for the interviewer).
- I have a list of topics that I want to address. Please remember that there are no right or wrong answers. Feel free to pause and/or ask questions at any stage during the interview. I might make a few notes in case I want to come back to something later.
- The discussion should not take more than 60 minutes.
- Is it okay if I audiotape this interview today?
- Do you have any questions?

Explain the task to the participant:

- The purpose of this interview today is to explore intervention options and gather your feedback on the proposed content, features and appearance of DARTT.
- I would also like to ask your advice on how the proposed intervention could be improved to make it more acceptable and suitable for clinical practice.
- Everything you tell me will be kept confidential. To protect your privacy, I won't connect your name with anything that you say or provide any information that might identify you.

Using a PowerPoint presentation, each interview will begin with a brief description of the intervention components. Guided by the RE-AIM Framework, the participant will then be asked the following questions, as well as any questions related to specific points raised during the presentation:

1. REACH
• What group of healthcare professionals do you think the intervention will appeal to and why?<br>• In your opinion, would your colleagues find the proposed intervention acceptable and practical? Why (not)?
**2. EFFECTIVENESS**
• What was your initial impression of the intervention?<br>• Would it be suitable for clinical use? Why?<br>• Was there anything you didn`t like or that you expected to see but didn't (i.e., strengths and weaknesses)?<br>• Which intervention elements do you see as the most important? Why?<br>• Can you think of any issues that would prevent it from working?<br>• Can you think of other similar interventions that have worked in a hospital setting? Elaborate.<br>• One of the intervention components is a feedback mechanism. How do you think people will respond to individual feedback? What kind of feedback on prescribing is acceptable and who do you think should provide it?<br>• What is the most important outcome you expect to see using this intervention? Why is it important to you?
**3. ADOPTION**
• What settings do you think the intervention would be the most suitable for (i.e., across acute hospitals, only medical units)? Why?<br>• What training or support, if any, do you think hospital prescribers would need to use the intervention?<br>• What would be the most important elements of training?
**4. IMPLEMENTATION**
• How do you think the intervention should be delivered?<br>• Can you think of any adjustments or modifications that would strengthen or improve it to increase its uptake and effectiveness?<br>• Do you think it will work in real practice?<br>• Can you think of any challenges we will need to overcome to implement this in 'real world' practice?
**5. MAINTENANCE**
• How can we ensure the intervention is sustainable over time?<br>• What factors need to be in place, and what needs to be avoided/minimised?<br>• How do you think we should track success and feedback about the intervention?

End of interview

Is there anything else that you would like to add about any of the topics that we've discussed or other areas that we didn't discuss but you think are important?

Thank you for your time and participation in this interview. The information that you provided to us will be very helpful in this project.

Consolidated criteria for reporting qualitative research (COREQ): a 32-item checklist for interviews and focus groups

No Item	Guide Questions / Description	Details / Reported on page no
**Domain 1: Research team and reflexivity**		
**Personal Characteristics**		
1. Interviewer/facilitator	Which author/s conducted the interview or focus group?	First Author
2. Credentials	What were the researcher's credentials? *e.g., PhD, MD*	First Author: BSc, MSc, PhD, FHEA<br>Second: PhD, MSc, BSc, Dip HV, RN, SFHEA<br>Third: PhD, MSc, MA, RN, FHEA<br>Fourth: PhD, Cpsych, AFBPsS, RN, SFHEA<br>Fifth: PhD, BSc<br>Last: PhD, MSc, BSc, SFHEA
3. Occupation	What was their occupation at the time of the study?	First Author: Lecturer and PhD scholar<br>Second: Professor of Nursing<br>Third: Senior Research Fellow<br>Fourth: Senior Lecturer<br>Fifth: Professor in Behavioural Science, Head of Research Development<br>Last: Senior Lecturer
4. Gender	Was the researcher male or female?	The research team consisted of four females and two males. The main researcher (first author) is female.
5. Experience and training	What experience or training did the researcher have?	The research team consisted of five active faculty members (professors or lecturers), all with white backgrounds (four British and two European). The first author was a PhD student at the time of data collection and analysis, and the study was part of her doctoral work. She had previous experience in coordinating clinical trials and academic studies and received training in data collection and analysis. The team had substantial expertise in qualitative research methods, with professionals from nursing, psychology, and social science backgrounds, focused on developing healthcare interventions. The research was conducted in close collaboration with an NHS hospital health boards and trusts, with advisory input from clinicians (microbiology and infectious diseases) and a behaviour change expert.
**Relationship with participants**		
6. Relationship established	Was a relationship established prior to study commencement?	Partially. In her previous clinical role as a Critical Care Research Coordinator, the first author/interviewer had met four participants.
7. Participant knowledge of the interviewer	What did the participants know about the researcher? e.*g., personal*	Participants were informed about the study's purpose and acknowledged that it was a research project. Ethical approval was obtained, and participants

	goals, reasons for doing the research	reviewed detailed information before providing their written informed consent to participate
8. Interviewer characteristics	What characteristics were reported about the interviewer/facilitator? e.g., *Bias, assumptions, reasons and interests in the research topic*	Participants were aware that the researcher had a dual background in nursing and public policy and was also a PhD scholar.
**Domain 2: study design**		
**Theoretical Framework**		
9. Methodological orientation and Theory	What methodological orientation was stated to underpin the study? *e.g., grounded theory, discourse analysis, ethnography, phenomenology, content analysis*	This study is grounded in the philosophical standpoint of pragmatism, emphasising the adoption of practical methods. Guided by the MRC Framework for developing and evaluating complex interventions, it utilised a person-centred approach and incorporated behavioural science principles. Data were analysed using Framework Analysis.
**Participant selection**		
10. Sampling	How were participants selected? *e.g., purposive, convenience, consecutive, snowball*	A purposive, diverse sample of Healthcare Professionals (HCPs) and lay participants (health service users).
11. Method of approach	How were participants approached? e*.g., face-to-face, telephone, mail, email*	Professional recruitment included sending project information to specialist clinical networks, using NHS Intranet advertisements and internal emails. Targeted online approaches included using antibiotic-related Twitter (now X) pages/forums. The study was also listed on a certified research recruitment website. For lay participants, a gatekeeper strategy was used whereby an NHS Patient Advisory Service representative contacted potential participants after receiving the research protocol. Interested volunteers received a Project Information Sheet from the lead researcher with a follow-up email sent if there was no response within three working days. Phase 1 participants who had previously expressed interest in taking part in future research were also directly contacted and invited to interviews (Phase 2). Several participants also forwarded study details to their colleagues interested in antimicrobial stewardship. Participants received no financial incentive.
12. Sample size	How many participants were in the study?	Diverse sample of 15 HCPs with varying experience levels and 3 lay participants were recruited.
13. Non-participation	How many people refused to participate or dropped out? Reasons?	Out of 24 participants who initially expressed interest, 3 did not respond to the follow-up email, and 1 cancelled the interview due to clinical work commitments. Data saturation was reached after 18 interviews, and recruitment ceased. None of the 18 participants who consented and participated withdrew.
**Setting**		

14. Setting of data collection	Where was the data collected? e.*g., home, clinic, workplace*	The interviews were conducted during the COVID-19 pandemic, so data collection took place online via Microsoft Teams. Most participants joined from home, while some HCPs joined during clinical hours from their offices.
15. Presence of non-participants	Was anyone else present besides the participants and researchers?	No
16. Description of sample	What are the important characteristics of the sample? *e.g., demographic data, date*	Participant characteristics are reported in the main manuscript in Table 2 (Section 4.3).
**Data collection**		
17. Interview guide	Were questions, prompts, guides provided by the authors? Was it pilot tested?	Yes, the researcher used a Topic Guide (see Supplemental Material, page 2), which was pilot tested before use.
18. Repeat interviews	Were repeat interviews carried out? If yes, how many?	No
19. Audio/visual recording	Did the research use audio or visual recording to collect the data?	Data were audio-recorded using a Digital Voice Recorder, which was encrypted to the AES256 standard.
20. Field notes	Were field notes made during and/or after the interview or focus group?	Yes, the interviewer made field notes after the interviews.
21. Duration	What was the duration of the interviews or focus group?	The interviews lasted 45–60 minutes.
22. Data saturation	Was data saturation discussed?	Yes, data saturation was reached after 18 interviews, as no new themes emerged, and recruitment ceased.
23. Transcripts returned	Were transcripts returned to participants for comment and/or correction?	No
**Domain 3: analysis and findings**		
**Data analysis**		
24. Number of data coders	How many data coders coded the data?	One researcher (first author/interviewer) coded the data, while the second researcher (last author) conducted accuracy checks on a sample of approximately 20% of the dataset (4 transcripts), with ongoing input from the research team.
25. Description of the coding	Did authors provide a description of the coding tree?	Data from the interviews were analysed using the five stages of framework analysis: familiarisation with the data, developing a theoretical framework, indexing, charting, and mapping/interpretation.<br><br>Initially, the researcher familiarised hers with the data by transcribing and reviewing the interviews, noting key ideas and themes. A thematic framework was then identified using a 'hybrid' approach, combining deductive and inductive coding. The study's focus on

		the intervention's acceptability and usability guided the framework, starting with predefined codes based on the RE-AIM Framework. After testing the codes on two transcripts and discussing them with academic supervisors, categories like 'reach' and 'effectiveness' were modified to include 'acceptability' and 'usability' for greater clarity.<br>The initial coding was done by two researchers (first and last author), and after reviewing the first four transcripts, a final set of codes was agreed upon. These codes were grouped into six themes, as shown in the Coding Index (page 12).<br>In the indexing stage (page 13), each transcript was applied to the framework. The data were organised in a matrix, summarising the coded transcripts. During the charting stage, the indexed data were rearranged into a chart for clearer visualisation, preserving the original meaning. The chart was organised by headings and subheadings from the Form of Delivery framework (page 18).<br>In the final stage, mapping and interpretation, connections between categories were explored through a schematic diagram (page 19), enabling a deeper understanding of the research issues.
26. Derivation of themes	Were themes identified in advance or derived from the data?	To translate the planned intervention into practice, our data analysis was guided by a *priori* issues identified using the RE-AIM (Reach, Effectiveness, Adoption, Implementation, and Maintenance) Framework, commonly used to adapt interventions. However, we remained open to new and unexpected concepts. For example, the initial 'reach' and 'effectiveness' categories from the RE-AIM Framework did not fully capture participants' likes and dislikes of the intervention. These categories were modified, and new codes reflecting user language and experiences were created. Data analysis was iterative, with disagreements resolved through consensus.
27. Software	What software, if applicable, was used to manage the data?	Yes, NVivo (Version 12) qualitative software was used to facilitate data analysis.
28. Participant checking	Did participants provide feedback on the findings	There were five iterative interview cycles, with the research team making changes to the intervention after each cycle and the interviewer presenting them to the next group of participants for feedback. This process was repeated four times to refine and optimise the prototype intervention.
**Reporting**		
29. Quotations presented	Were participant quotations presented to illustrate the themes / findings? Was each quotation identified? e*.g., participant number*	Yes – see the Results section in the main manuscript.

30. Data and findings consistent	Was there consistency between the data presented and the findings?	Yes
31. Clarity of major themes	Were major themes clearly presented in the findings?	Yes, four key themes emerged, which are described in the main manuscript.
32. Clarity of minor themes	Is there a description of diverse cases or discussion of minor themes?	Yes, cases that refuted the key findings are described in the main manuscript.

Digital Antibiotic Review Tracking Toolkit (DARTT): Component Summary developed in Phase 1

Component 1					
**Format**	**Content**	**Delivery**	**Incorporated BCTs**	**Mechanism of action**	
				↑ COM-B	TDF
**Digital Antibiotic Review Tracker** (+ feedback on performance)	Professionally designed CDSS for electronic implementation in clinical practice, including:<br>• User-friendly interface<br>• A log of the user`s last activity<br>• Individual dashboards with a menu of outstanding actions<br>• Antibiotic time-out traffic-lights reminder system<br>• A pop-up window with the summary of microbiology results<br>• Creates trail of decisions<br>• Allows generation of reports for antibiotic timeout-eligible patients<br>• Basic logic tree for correct dosing regimens, including online calculator<br>• Instant access to the Antibiotic Companion app<br>• Creates individual performance reports<br>• Prompts discussion with the clinical team and the patient<br>• Infectious diseases input required for continuation of restricted broad-spectrum therapy (as per AWaRe classification)<br>• Hyperlinks embedded to online resources (e.g., antibiotic guidelines)<br>• Monthly individual and area reports on antibiotic-prescribing practice<br>• The reports will include aggregated data for the number of initiated antibiotics,	The tracker will be integrated into the electronic record system, accessible on NHS-enabled devices and delivered at the point of care.<br><br>The interface will run on all internet browsers.<br><br>Delivered by email to prescribers and	Goal setting (behaviour); Problem solving; Action planning; Feedback on behaviour; Feedback on outcome of behaviour; Social support (unspecified); Instruction on how to perform the behaviour; Information about health consequences; Information about social and environmental consequences; Information about emotional consequences; Discrepancy between current behaviour and goal; Social comparison; Prompts/cues; Behavioural practice/ rehearsal; Habit formation; Habit reversal; Credible source; Reduce negative emotions; Verbal persuasion about capability; Focus on past success; negative emotions; Restructure the physical environment; Restructuring the social environment; Add objects to the environment	Psychological Capability; Physical Capability; Psychological Capability; Physical Opportunity; Social Opportunity; Reflective Motivation; Automatic Motivation	Skills; Knowledge; Behavioural regulation; Memory, attention and decision processes; Environmental context and resources; Social influences; Beliefs about capabilities; Beliefs about consequences; Goals; Reinforcement; Emotion

	streamlined prescriptions and incomplete/incorrect information<br>• Presented as a table and/or bar chart in a PDF file<br>• Comparison with peers<br>• Accompanied by a narrative with links to antibiotic guidelines	discussed with the 'clinical champion'.<br><br>Local area reports encouraged to be discussed in team meetings.			
**Component 2**					
**Format**	**Content**	**Delivery**	**Incorporated BCTs**	**Mechanism of action**	
				↑ COM-B	TDF
**Webinar** (recorded and/or live stream)	Professionally delivered by a relevant practicing hospital clinician (e.g., a member of local AMS team, infection control specialist or microbiologist), lasting no more than 20 min, incorporated into staff meetings and summarising:<br>• Importance of AMR & active time-out<br>• Introduction to DARTT<br>• Safety of stopping/de-escalating antibiotics<br>• Local AMR data<br>• Promote action planning<br>• No increase in workflow<br>• Potential benefits of DARTT	Webinar delivered through an electronic link embedded in the DARTT supporting documentation<br><br>Clinicians offered a live stream option and encouraged to present the Webinar in staff meetings	Goal setting (behaviour); Problem-solving; Information about health consequences; Information about social and environmental consequences; Information about emotional consequences; Credible source; Reduce negative emotions; Focus on past success	Psychological Capability; Social Opportunity; Reflective Motivation; Automatic Motivation	Knowledge; Social/professional role and identity; Social influences; Beliefs about capabilities; Beliefs about consequences; Goals; Emotion
**Component 3**					
**Format**	**Content**	**Delivery**	**Incorporated BCTs**	**Mechanism of action**	
				↑ COM-B	TDF
**E-training**	Evidence-based educational e-learning activity for self-completion to increase motivation and competence to review	HCPs will be sent a weblink to the e-learning activity, which will be	Action planning; Problem-solving; Discrepancy between current behaviour and goal; Instruction on how to	Physical Capability; Psychological Capability;	Skills; Knowledge; Behavioural regulation; Memory, attention and decision processes;

	antibiotics, lasting no more than 10-15 min, and including:<br>• Background on AMR<br>• The role of HCPs in antibiotic time-out<br>• Demonstration of DARTT tools<br>• 2 interactive, animated clinical scenarios of a consultation by a prescriber with an adult patient<br>• Emphasis on fostering optimal prescribing behaviours at time-out<br>• Encourages prescribers to ask question about antibiotics using the '3+3' format (what, why, how long and review therapy after three days).<br>• Individual feedback given<br>• Training incorporated into induction<br>• CPD certificate provided after completing the e-learning	accessible on any internet-enabled device.<br><br>Up to 3 email and text reminders will be sent to complete the activity.	perform the behaviour; Information about health consequences; Information about emotional consequences; Demonstration of the behaviour; Social comparison; Identification of self as a role model; Prompts/cues; Behavioural practice/ rehearsal; Habit formation; Habit reversal; Credible source	Physical Opportunity; Social Opportunity; Reflective Motivation; Automatic Motivation	Environmental context and resources; Beliefs about capabilities; Beliefs bout consequences; Social/professional role and identity; Emotion
**Component 4**					
**Format**	**Content**	**Delivery**	**Incorporated BCTs**	**Mechanism of action**	
				↑ COM-B	TDF
**Patient information materials (PIMs)**	Professionally designed PIMs, including:<br>• Patient leaflet to improve patient knowledge and enhance shared decision-making<br>• Presented in a double-sided A4 format<br>• Explaining the risks of antibiotics (i.e., AMR and side effects) and the importance of a timely re-assessment, stopping or narrowing down antibiotics<br>• Poster encouraging patients to ask HCPs about antibiotics	Patient information leaflets and posters placed in the hospital waiting rooms provided in a health literate format.	Instruction on how to perform the behaviour; Information about health consequences; Information about social and environmental consequences; Information about emotional consequences; Prompts/cues; Habit formation; Reduce negative emotions; Add objects to the environment	Psychological Capability; Physical Opportunity; Reflective Motivation; Automatic Motivation	Knowledge; Environmental context and resources; Beliefs about capabilities; Beliefs about consequences; Emotion

Coding Index

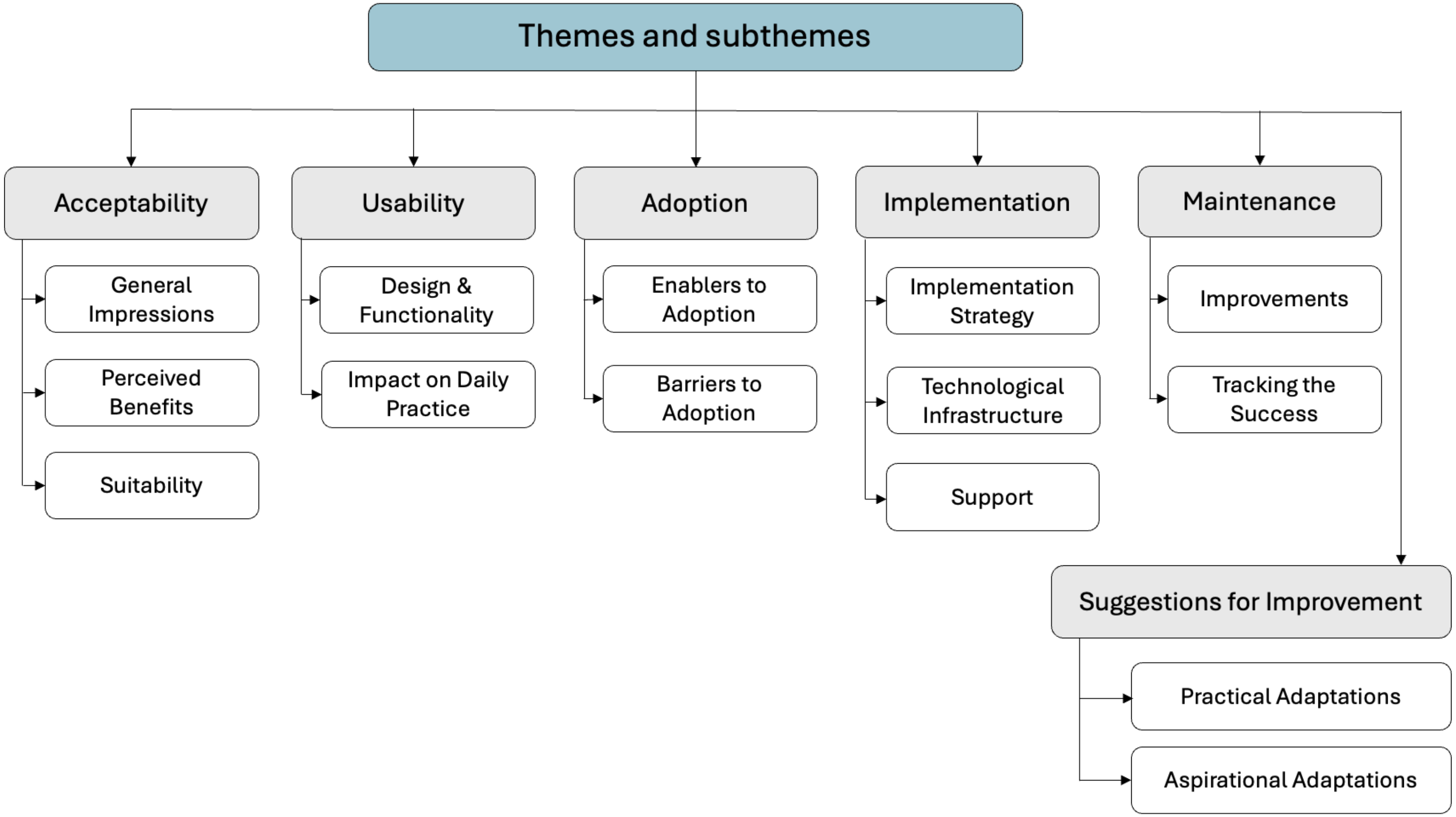

Indexing Example: Thematic Categorisation of Data

No	Acceptability	Usability	Adoption	Implementation	Maintenance	Suggestions for improvement
**P01**	**1.1** Thinks DARTT is a great idea. 1<br>**1.1** Likes the idea of the Tracker being part of daily review. 2<br>**1.1** Feedback on prescribing would be great because there is very little information available on antibiotic hospital consumption. 12<br>**1.1** Likes the idea of having aggregated data so everyone can see what other areas order and use.16<br><br>**1.2** Thinks that the Tracker will create a trail of decisions. 28<br>**1.2** DARTT will make a big difference once we can electronically prescribe.<br>**1.2** The expected outcomes will be different for wards and ITUs. 32<br><br>**1.3** DARTT would work best in ICU because a formal round is done there every day, and it could become part of that standard round. 64<br>**1.3** It will appeal to ANPs because prescribing it's such a new skill for them. They`ll appreciate all the cues on the Tracker. 65<br>**1.3** The tracker is the key component, but the Webinar and training are also needed. 76<br>**1.3** The problem with feedback is getting people to pay attention to the feedback. Individual emails	**2.1** The Tracker has to be intuitive and tell prescriber what they should be thinking, reminding to do the right thing. 32<br>**2.1** The Antibiotic 3+3 will give nurses a script of questions to asks the prescribers and prompt them to review. 34<br>**2.1** PIMs are available, people tend just to see them and not pick them up, but you do need something for families, especially in critical care. 36<br><br>**2.2** The Tracker shouldn`t stop HCPs from doing what they intended to do because it then creates a workflow problem. 74<br>**2.2** Everybody knows TRAK, so DARTT potentially will just flow as part of the new way of doing it. 77	**3.1** Approach Directorates and talk to them about the product to gain advice and support. 38<br>**3.1** Training is required to make sure that people can practice.42<br><br>**3.2** Thinks that there are always people who just won't engage regardless of how DARTT will be rolled it out but getting engagement first will help. 66	**4.2** Technology will be a problem because it`s really difficult in the NHS and some computers are 20 years old. 18<br>**4.2** Relying on Wi-Fi in the NHS is a problem as the upload speed is very slow. 20<br>**4.2** Reflect on the experience of making family videos for patients during COVID and poor internet coverage. 22<br>**4.2** The interface will require NHS coverage for they may allow better security, but the problem is the IT security in the NHS. 24<br>**4.2** Having Kardex and electronic prescribing would not be practical.<br>**4.2** Doesn`t think that pop-up reminders would be easy to install on TRAK. 39<br><br>**4.3** Accessibility of the computers at the bedspace. 55	**5.1** Talks about maintaining the system similar to TRAK and doing upgrades when necessary. 41<br><br>**5.2** The feedback has to show a clinical benefit to using DARTT. 43<br>**5.2** Thinks that tracking success will be easy as the Tracker will generate data on de-escalation and prescribing according to guidelines. 63	**6.1** Would like to have more nurse involvement in ITUs and thinks that Antibiotic 3+3 will help with that. 17<br>**6.1** It would be practical to link the Tracker with microbiology reviews so that microbiologists could review antibiotics remotely.19<br>**6.1** Thinks the Tracker should be made mandatory to be effective. 21<br>**6.1** Suggests that only microbiology should have privileges to override the system. 29<br>**6.1** Training could be incorporated into Learn-pro. 31<br>**6.1** Training shouldn't be a big, huge, elaborate thing. 31<br>**6.1** Training should focus on allowing HCPs to practice using DARTT. 35<br>**6.1** Suggests placing `Antibiotic 3+3` sticker on the antibiotic prescription page, nursing care plans, daily medical reviews, or on TRAK to trigger a review. 37<br><br>**6.2** Would like the system to connect to the patient clinical picture, so people are guided through the de-escalation review. 59<br>**6.2** Suggest creating pop-up messages or outstanding action

	generated by the Tracker would solve this issue. 79<br>**1.3**. It would be acceptable to put the Antibiotic 3+3 sticker on nursing care plans or in the daily medical reviews, or even on TRAK to make people go to the Tracker. 83<br>**1.3** Believes that the intervention would be transferable to GP practices. 91					reminders with recommendations from microbiology. 61<br>**6.2** Suggests inbuilding a calculator for specific antibiotics, such as Vancomycin and Gentamicin. 69<br>**6.2** Thinks that there has to be instant access to the MicroGuide. 71<br>**6.2** Default option needed for antibiotics that aren't bug-related (e.g. Erythromycins for gut motility) that automatically disables the 3-day reminder. 77
**P12**	**1.1** The intervention looks great and visually appealing. The imagery is beautiful; it's quite impactful, it's simple, it looks user-friendly, straightforward, the components are all very sensible, it's got clear clinical value. 5<br>**1.1** Can normally be quite picky but can`t see anything negative. 6<br>**1.1** Likes the idea of having aggregated data – it would be helpful for ANPs` yearly PDPs, evidence for their re-validation, for their portfolio. 6<br><br>**1.2** Thinks that DARTT will help to rationalise antibiotics. 12<br><br>**1.3** Timing is good because of the HEPMA system being rolled out. 14<br>**1.3** Convinced that all the ID consultants and microbiologists will love the intervention. 16	**2.1** Data on antibiotic use at the unit level helps show local differences and identify disparities and outliers. 53<br>**2.1** Re online training, thinks that some multiple-choice questions can be useful afterwards to see what has been learned. 61<br>**2.1** Unsure about the value of interactive online learning as well as a video. 62<br>**2.1** Live-streamed video is really good in terms of interaction because you can get people on board, but it is difficult in terms of resources.65	**3.1** If people can find a workaround that they perceive is quicker; they will do it.42<br>**3.1** The tracker has to be straightforward and simple to use and as least clunky as possible. 42<br>**3.1** Can`t think of anybody in particular who would be resistant to it. 45<br>**3.1** Thinks that there will always be resistance to change, but DARTT is pushing against open doors. 46<br>**3.1** Engagement from the top-down and people on the floor is	**4.1** Talks about previous experience of implementing intervention and the importance of going round the wards and hospitals and talking to people about it. 30<br>**4.1** Start in the places that will use it the most and get people familiar with it, where people are prescribing lots of antibiotics. 84<br>**4.1** Talks about getting more junior doctors using it in a small area 86<br>**4.1** Suggest trialling DARTT in ID and AMU first, where antibiotic prescribing is a big part of clinical practice. 91<br>**4.1** Suggest rolling DARTT out with new doctors starting. 92	**5.1** It`s important to change things and improve the system. 52<br>**5.1** Talks about the importance of easing out the glitches as quickly as possible and adjusting DARTT based on real-life users' feedback. 52<br>**5.1** Talks about being open to change. 53<br><br>**5.2** The outcomes will be hard to measure or quantify because nobody's measuring it pre-intervention. 141	**6.1** Reflects on recent online HEPMA training. Suggests that online video training helpful and useful and see the functionality of the system. 113<br>**6.1** Once the intervention is out into a hospital; it could be part of an induction for the new doctors. 116<br>**6.1** Live Webinar is really good in terms of interaction and getting people on board but suggests that live-streamed is not necessary once it`s out into a hospital. 137

	**1.3** Doesn`t think posters are effective, describes `poster blindness`. 18<br>**1.3** The tracker is the most important but can`t be achieved without the webinar or interactive tool. 21<br>**1.3** PIMs are the least important; it`s nice, but more of a bonus add on that could fall out of the others. 24<br>**1.3** Points out that hospital antibiotic prescribing is very different from GP practice as there`s space for discussion due to illness acuity. 31<br>**1.3** Thinks that the dialogue with the patients is important, but patients just want someone to make them feel better. 35<br><br>**1.3** Felt uneasy about the text message reminders. 46<br>**1.3** Feedback is really important, but different HCPs have different feedback requirements. 49<br>**1.3** Feedback necessary for ANPs because they have to provide evidence of their prescribing and reflection on that as opposed to consultants. 50<br>**1.3** Wouldn`t like comparison with colleagues. 52<br>**1.3** Suggests that the aggregated data would also work for GPs as it's helpful for them looking at their finances. 56	**2.2** If DARTT takes extra time, HCPs need to perceive the real benefits that make it worthwhile. 72	required for uptake. 49<br>**3.1** Enthusiasm and buy-in are required to spread the word about the intervention. 52<br>**3.1** Thinks training is required, but it can`t be burdensome. Undecided whether training should be mandatory or voluntary. 60<br>**3.1** Thinks that the Webinar and interactive stuff will be really helpful when first getting DARTT out to advertise it, raise awareness and get buy-in. 61<br>**3.1** Junior doctors are great for spreading knowledge about new stuff.67<br>**3.1** Junior doctors are positive promoters as they move around and between Health Boards. 67<br><br>**3.2** DARTT has to be as user friendly as possible and not add unnecessary or burdensome steps as junior doctors will find workarounds. 81	**4.1** Not a fan of local champions but thinks that people listen to positive influencers - important to engage with to ensure successful implementation. 96<br>**4.1** Successful implementation depends on preparation and getting the high-level people buy-in, also the TRAK people and HEPMA people buy-in to make it work, so it's not clunky. 97<br>**4.1** Hard graft has to be put in during the initial roll-out. 104<br>**4.2** Thinks DARTT would be able to slot into one of the version updates for HEPMA. 112<br>**4.2** Moving between paper and electronic would be quite annoying.119<br><br>**4.3** Pharmacy support required with rollout out of the DARTT. 120<br>**4.3** Having specific individualised support would be really helpful. 120<br>**4.3** Thinks that implementation will be easy as everyone knows about antimicrobial stewardship, 129		

**P16**	**1.1** Thinks that DARTT is a great idea, looks very logical, everything is interlinked, is easy to follow and understand, and is an excellent package. 8<br>**1.1** Can`t think of anything missing. 8<br>**1.1** Likes the ambition to fully integrate this so that it dovetails with everything that's happening with the patient. 10<br>**1.1** Likes that DARTT considers various people at all the various stages likely to be involved in monitoring and administering antibiotics. 10<br>**1.1** Likes that the interests of the patients have been considered. 11<br>**1.1** Felt uneasy about people being compared to colleagues as they might be put off, and it will reduce the uptake. 24<br>**1.1** Reflects that sometimes the best doctors have the most fatalities because they are prepared to take on difficult cases. 26<br><br>**1.2** Believes that DARTT will help integrate patient care between different clinical teams and areas and close the communication gap. 32<br>**1.2** Anticipates that the Tracker will stop any unintended continuation of antibiotics and give HCPs closer focus on how the patient responds to treatment. 34	**2.1** The leaflet is simple. It`s prompt the patient to do a bit more. It wouldn't put a patient off but reassure/indicate what the next steps might be. 29<br>**2.1** Poster is useful and ties in with the leaflet in terms of the overall strategy. 32<br>**2.1** The poster gives the patient a script of questions to ask. 33<br>**2.1** Likes the idea of the Tracker generating daily reports the creating collective responsibility for antibiotic review. 38<br>**2.1** Compares DARTT to a one-stop-shop for clinicians to go in and think about the patient's progress. 41<br>**2.1 S**imple messages such as the biotic 3+3 are the most effective. 53<br><br>**2.2** The Tracker will encourage HCPS to speak to somebody who maybe knows a bit more or check if they are doing the right thing. 56	**3.1** People need to see DARTT as a priority to buy into it. 36<br>**3.1** Need to win over people who would give it a fair go and provide honest feedback. 38<br>**3.1** Engagement with patients is key because that will help to endorse the value of the whole project. 40<br>**3.1** DARTT will need support at very senior levels and an endorsement to say that this is worth doing. 40<br><br>**3.2** Thinks that there is always some resistance to anything new. 66<br>**3.2** Reassurance will be required for those HCPs who feel that this is more work. 68	**4.1** Some big data systems take a long time to implement, and phasing things can sometimes buy more time to better reflect on other parts of the project's implications. 52<br>**4.1** Once the system is officially proven, it could be rolled out on a more wide scale. 54<br>**4.1** To roll out DARTT Scotland-wide, gatekeepers for medicines in every Health Boards are needed to take an overall responsibility (i.e. champions, guardians or clinical pharmacists). 54<br>**4.1** Reflects on previous experience of setting up software and points out the need to get the expertise and the money to set DARTT up. 56<br>**4.1** Suggests finding a willing partner in the first phase. 56<br>**4.1** An experienced group of people responsible for implementation is vital. 58<br>**4.1** Suggest doing a pilot. 112<br>**4.1** Suggests starting implementation with the webinar and the online interactive tool and then testing the Tracker in one Health Board, maybe in one	**5.1** The system needs to evolve continually. 130<br>**5.1** Improving the software based on feedback is important. 132<br><br>**5.2** Suggests looking at the impact that PIMs will have and how effective one particular type of communication is over another. 146<br>**5.2** The cost-benefit need to be looked at to track the successes of DARTT. 159	**6.1** Suggests looking at the WHO reports on AMR and drawing on that for PIMs. 2<br>**6.1** Recommends introducing DARTT as a professional development opportunity for junior prescribers. 61<br>**6.1** Suggests introducing DARTT training to junior doctors during their undergraduate training. 62<br>**6.1** Training should also be provided when new staff begin.63<br>**6.1** Suggest inbuilding the `Antibiotic 3+3`reminder into the Tracker software. 82

	**1.3** DARTT would work best in general wards as staff have a far broader role, far bigger number and range of patients and tasks. 49<br>**1.3** The Tracker and the Antibiotic 3+3 prompt are the most important elements of DARTT. 51<br>**1.3** A review of professional performance has to be dealt with sensitively because of the variation in prescribing. 73<br>**1.3** The hierarchy of authority within hospitals is quite sensitive, so the feedback has to come from an HCP whose qualifications are senior to that person, someone who has authority, position and is highly respected, a champion or a clinical pharmacist who's sufficiently knowledgeable.74<br>**1.3** Points out that the `Antibiotic 3+3` can`t be overly intrusive and pop-up reminders too frequent. 81<br>**1.3** Likes the idea of placing a poster in the waiting area. 85 **1.3** Simple, straightforward messages are good for raising awareness. 85<br>**1.3** Reflects on his own experience of sitting in the waiting room and wanting to take in information. 85<br>**1.3** Highlights that if people don't get an answer to what they've been alerted to, it can cause more concern if they're already distressed. 89<br>**1.3** If expectations are raised, you have to ensure that they are being met. Otherwise, the patient and their relatives will become more unsettled. 90	**2.2** DARTT mustn't add additional work but streamline it. 101		ward. PIMs can be left till last. 113<br>**4.1** Talks about the importance of addressing and pre-emptying any difficulties before the implementation. 129<br><br>**4.2** The Tracker needs to be compatible with electronic health records. 137<br>**4.2** Need to assess the feasibility of the development of the Tracker and what that would mean and what the proforma would look like, how it would all tie together with the CDSS. 143<br>**4.2** Ease of linking the components is important. 149		

Charting

DARTT element or feature	Acceptable & feasible?	Suggestions and future considerations
**Provider**		
DARTT Team	A (+/-), F (+)	Project management team required; need to link to local AMS teams. Leadership and organisation-wide engagement essential. Suggestions to involve/offer project development opportunity to junior doctors and ANPs to champion DARTT.
**Delivery Format**		
Webinar	A (+), F (+/-)	Resources required for multiple delivery of live Webinar; pre-recorded version acceptable and more feasible.
Online Interactive Tool	A (+), F(+/-)	Online and mandatory format acceptable. Long training not feasible in a busy hospital setting. Preference for a short video demonstrating how to use the Tracker and a short practical session. Training embedded into induction packages and ward education acceptable. Suggestions for easy access (e.g. accessible at home).
Antibiotic Review Tracker	A, F (+/-)	Only feasible in areas with electronic prescribing. Need to consider the technical infrastructure available and system compatibility.
Feedback	A, F (+/-)	Regular face-to-face feedback not acceptable and not feasible to be delivered to a large number of staff. Preference for automated emails with aggregated data. Individual feedback only acceptable at annual appraisals or when practice issues identified. Unit- and hospital-level feedback and comparison acceptable.
\`Antibiotic 3+3\` reminder	A (+/-), F (+)	Acceptable and feasible as part of the Tracker, NHS banners, posters, stickers, emails. Poster \`blindness\` and frequency of reminders may be an issue – need to clinical importance, otherwise the risk of \`reminder fatigue\`. Text messages not acceptable. The feasibility of inbuilding it into the TRAK system has to be explored further. Preference for placing the reminder at bedspaces.
Patient Information Materials (PIMs)	A (+), F (+)	Posters and leaflets acceptable and feasible. If the patient/family is prompted to ask about antibiotics, ensure all staff prepared/trained to have those conversations.
**Materials**		
DARTT manual	A, F (+)	Preference for an online version, suggestions to provide flow charts and inbuild help boxes within the Tracker.
CPD certificate	A, F (+)	Particularly welcomed by junior doctors & ANPs – useful for their portfolio as evidence of learning.
**Setting**		
Acute hospitals	A, F (+)	Applicable to all hospital areas and potentially transferable to primary care.
**Intensity**		
1 hr long Webinar	A, F (-)	Not feasible to have 1 hr long Webinar – needs to be shorter to be incorporated into team meeting (e.g. 20 min).
Monthly discussions	A, F (+)	Monthly team discussions around the Tracker-generated prescribing reports acceptable
3-day review reminders	A (+), F (+/-)	If a prescription changed within 24 hours, the need to ensure the 3-day review trigger is automatically recalculated.
Support	A, F (+)	Regular contact with the DARTT team required; need to ensure availability of continuous technical support.
**Style**		
User-friendly interface	A, F (+)	Essential requirement.
Multiple data fields	A (-), F (+)	Need to minimise the number of data fields; preference for mandatory status.
System workarounds	A (-), F (+)	Workarounds not acceptable.

Mapping and Interpretation (conceptual map)

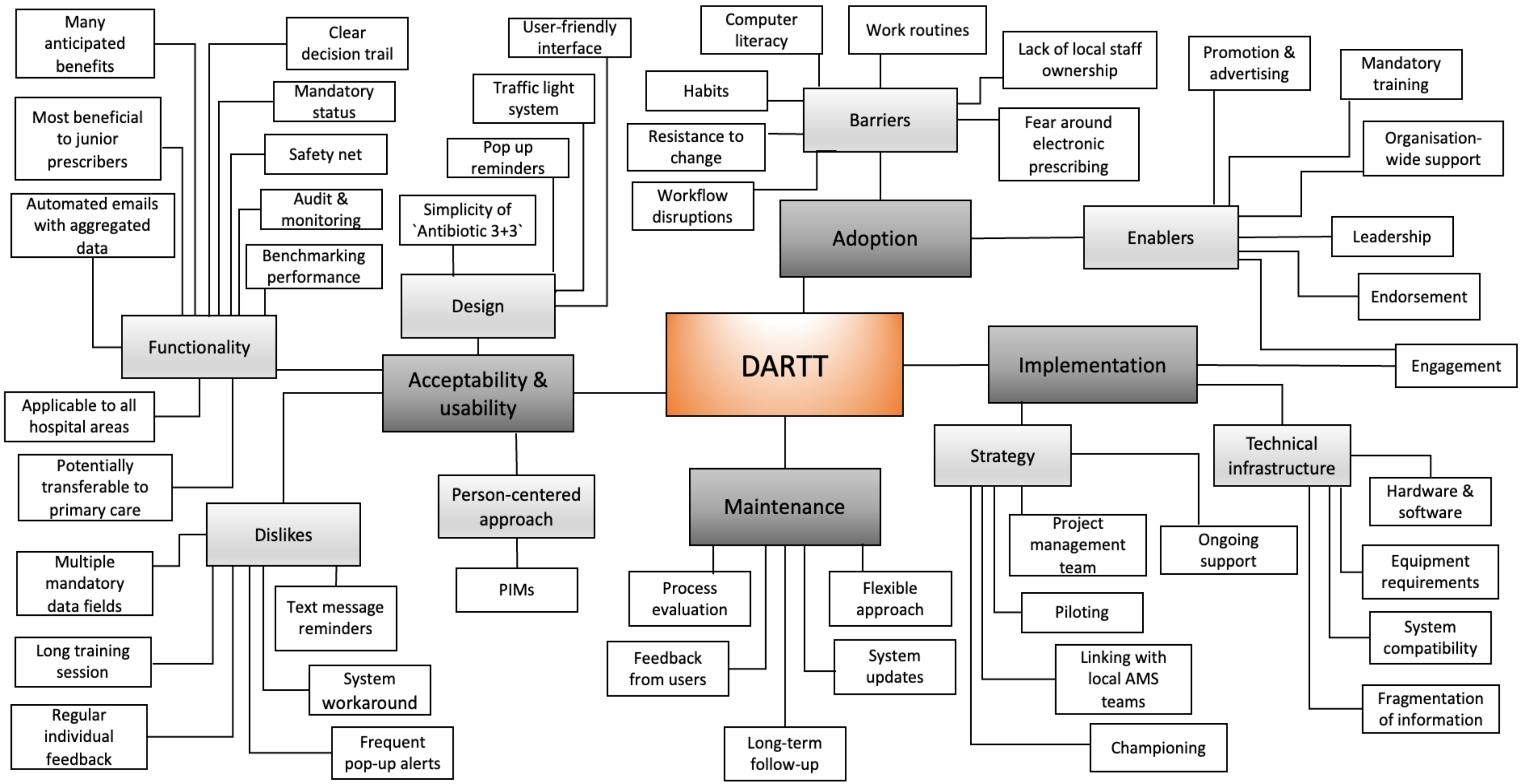

Supplement: Supplementary file 1 [file antibiotics-14-00660-s001.zip › antibiotics-3607369-supplementary.pdf]
